# Systemic Lupus Erythematosus-Associated Thrombotic Thrombocytopenic Purpura: A Case Report

**DOI:** 10.7759/cureus.85528

**Published:** 2025-06-07

**Authors:** Ounci Es-Saad, Adil Zyani, Ayman Bouchlaghem, Rajae Alkouh, Smael Labib

**Affiliations:** 1 Anesthesia and Critical Care, Mohammed VI University Hospital, Tangier, MAR; 2 Anesthesia and Critical Care, Faculty of Medicine and Pharmacy of Tangier, Abdelmalek Essaâdi University, Tangier, MAR

**Keywords:** adamts13, plasmapheresis, systemic lupus erythematosus, thrombotic microangiopathy, thrombotic thrombocytopenic purpura

## Abstract

Systemic lupus erythematosus-associated thrombotic thrombocytopenic purpura (SLE-TTP) is a rare but life-threatening condition that requires prompt recognition and treatment. We report a case of a patient with systemic lupus erythematosus (SLE) who presented with encephalopathy and was subsequently diagnosed with thrombotic thrombocytopenic purpura (TTP) based on ADAMTS13 (a disintegrin and metalloproteinase with thrombospondin motifs 13) deficiency. The patient was successfully treated with plasmapheresis.

A 42-year-old woman with a history of SLE presented with febrile encephalopathy and was admitted to the intensive care unit (ICU). Laboratory evaluation revealed microangiopathic hemolytic anemia and severe thrombocytopenia. MRI showed leptomeningeal enhancement and white matter changes suggestive of neuro-lupus. However, ADAMTS13 activity was <1% with detectable anti-ADAMTS13 antibodies, confirming the diagnosis of TTP. The patient received four sessions of plasmapheresis, high-dose corticosteroids, rituximab, cyclophosphamide, and supportive care. Following a steady improvement in consciousness, she was transferred to the internal medicine ward on day 17, with marked clinical and laboratory recovery.

This case highlights the importance of considering TPP in SLE patients presenting with acute neurological symptoms. Early recognition, prompt initiation of plasmapheresis, and immunosuppressive therapy can lead to favorable clinical and biological outcomes.

## Introduction

Systemic lupus erythematosus (SLE) is a chronic autoimmune disease with multi-organ involvement, ranging from mild mucocutaneous manifestations to life-threatening organ dysfunction. Hematologic manifestations of SLE include autoimmune hemolytic anemia, leukopenia, lymphopenia, and thrombocytopenia. Thrombotic thrombocytopenic purpura (TTP), while not a classical hematologic feature of SLE, may occur as a rare and severe complication. TTP is a subtype of thrombotic microangiopathy (TMA) that is exceedingly rare but potentially fatal [1,2].

TTP is characterized by thrombocytopenia, microangiopathic hemolytic anemia, and organ dysfunction. It is an autoimmune disorder mediated by antibodies against ADAMTS13 (a disintegrin and metalloproteinase with thrombospondin motifs 13), a von Willebrand factor-cleaving protease, leading to its severe functional deficiency [3]. Diagnosis is often based on the presence of microangiopathic hemolytic anemia and thrombocytopenia, with or without renal, neurologic, or fever-related symptoms. In clinical practice, tools such as the PLASMIC score are sometimes used to assess the likelihood of TTP, especially in settings where access to ADAMTS13 testing is delayed.

The overlap between SLE and TTP, referred to as systemic lupus erythematosus-associated thrombotic thrombocytopenic purpura (SLE-TTP), poses a significant diagnostic challenge due to the shared clinical features, including fever, renal involvement, and neuropsychiatric symptoms [4,5]. While primary (idiopathic) TTP is typically isolated, SLE-TTP occurs in the context of systemic autoimmunity, often complicating disease flares and presenting with overlapping laboratory and imaging findings. Although classically TTP was diagnosed clinically based on a pentad of findings, modern approaches rely heavily on laboratory confirmation, with an ADAMTS13 activity level <10% being diagnostic. Prompt initiation of plasma exchange and immunosuppressive therapy is essential for favorable outcomes [3,6].

This case highlights the diagnostic ambiguity between neuropsychiatric lupus and TTP in the setting of acute encephalopathy. It also underscores the importance of early ADAMTS13 testing and rapid initiation of combined immunosuppressive and plasma exchange therapy, which contributed to a favorable neurologic recovery.

## Case presentation

A 42-year-old woman with a known history of cutaneous and articular SLE, treated with hydroxychloroquine for one year, was admitted with febrile encephalopathy. She presented with jaundice, asthenia, and progressive altered mental status. On examination, she was febrile (38°C), hemodynamically stable, and had a fluctuating Glasgow Coma Score between nine and 12. Generalized jaundice and hepatosplenomegaly were noted.

Initial laboratory investigations were concerning for microangiopathic hemolytic anemia, evidenced by anemia, reticulocytosis, the presence of schistocytes on peripheral smear, elevated lactate dehydrogenase (LDH), and undetectable haptoglobin. The patient also had profound thrombocytopenia and elevated inflammatory markers, while renal function and electrolyte levels were within normal limits (Table 1).

**Table 1:** Initial hematologic and biochemical findings with their normal reference ranges MCV: mean corpuscular volume; MCH: mean corpuscular hemoglobin; MCHC: mean corpuscular hemoglobin concentration; LDH: lactate dehydrogenase; WBC: white blood cell; AST: aspartate aminotransferase; ALT: alanine aminotransferase; GGT: gamma-glutamyl transferase; HIV: human immunodeficiency virus

Parameter	Value	Reference Range
Hemoglobin	6.4 g/dL	11-14 g/dL
Hematocrit	18.9%	36-48%
MCV	108.8 fL	80-100 fL
MCH	37.1 Pg	27-33 pg
MCHC	34.1 g/dL	32-36 g/dL
Platelets	8000/uL	150000-400000/uL
Reticulocytes	446100/mm³	25000-75000/mm³
Schistocytes	2.6%	<1%
LDH	993 U/L	140-280 U/L
Haptoglobin	<0.1 g/L	0.3-2.0 g/L
Total WBC Count	17270/uL	4000-11 000/uL
Neutrophils	13310/uL	2000-7500/uL
Lymphocytes	3120/uL	1000-4800/uL
CRP	22 mg/L	<5 mg/L
AST	122 U/L	10-40 U/L
ALT	89 U/L	7-56 U/L
GGT	100 U/L	9-48 U/L
Alkaline Phosphatase	94 U/L	30-120 U/L
Total Bilirubin	3.8 mg/L	0.3-1.2 mg/dL
Direct Bilirubin	1 mg/L	0-0.3 mg/dL
Indirect Bilirubin	2.8 mg/L	0.1-1.0 mg/dL
Hepatitis A Serology	Negative	Negative
HIV	Negative	Negative
Sodium	141 mmol/L	135-145 mmol/L
Potassium	4.2 mmol/L	3.5-5.0 mmol/L
Chloride	100 mmol/L	98-107 mmol/L
Calcium	87 mg/L	85-105 mg/L
Vitamin B12	230 pg/mL	200-900 pg/mL
Folate (B9)	2.9 ng/mL	3-20 ng/mL
Urea	0.50 g/L	0.17-0.43 g/L
Serum Creatinine	7.7 mg/L	0.6-1.2 mg/dL
Coombs Test	Negative	Negative

**Figure 1:**
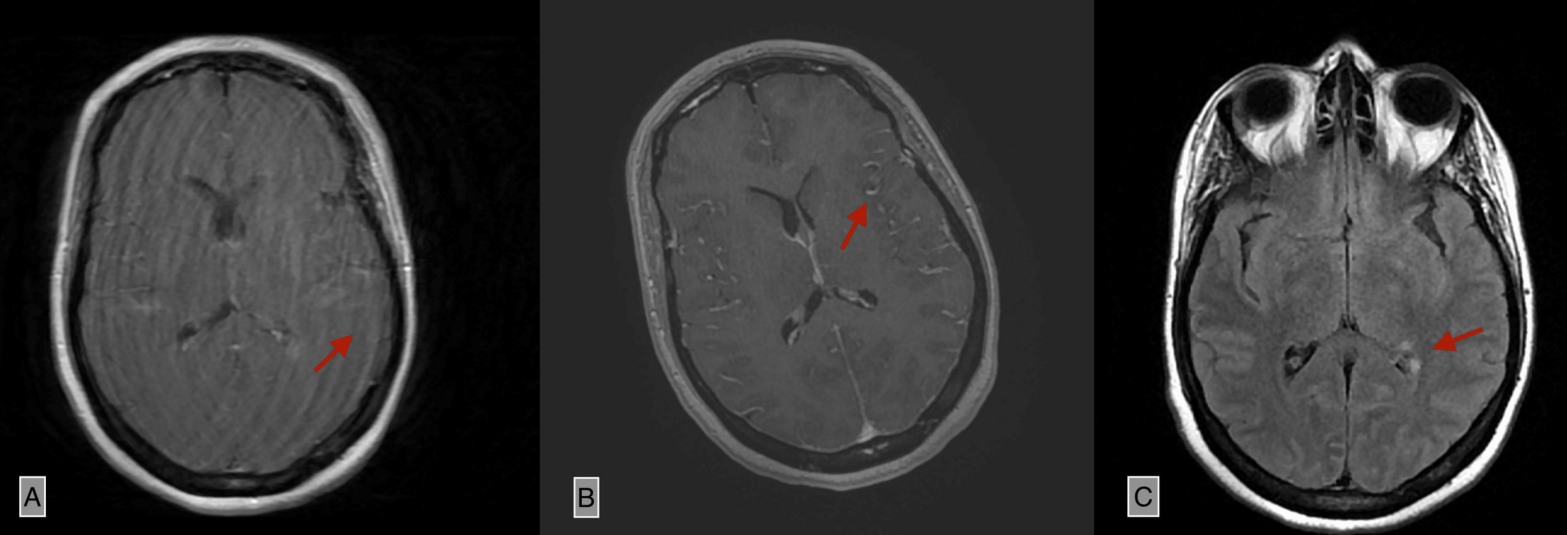
Contrast-enhanced MRI showing diffuse supratentorial leptomeningeal thickening and hyperintense signals in the left periventricular white matter, with post-contrast enhancement. These findings are suggestive of lupus-related vascular involvement with leptomeningitis (A) Axial T1-weighted post-contrast sequence showing periventricular contrast enhancement (arrow). (B) Axial T1-weighted post-contrast sequence demonstrating diffuse supratentorial leptomeningeal enhancement (arrow). (C) Axial FLAIR sequence showing periventricular hyperintense signal (arrow).

Cardiac evaluation by transthoracic echocardiography showed no abnormalities. Brain MRI revealed diffuse leptomeningeal enhancement and periventricular white matter hyperintensities with contrast enhancement, initially suggestive of neuropsychiatric lupus or CNS vasculitis (Figure 1).

The PLASMIC score was calculated at six, placing the patient in a high-risk category for severe ADAMTS13 deficiency. ADAMTS13 activity was subsequently found to be <1%, with positive anti-ADAMTS13 antibodies, confirming the diagnosis of acquired TTP. Autoimmune workup showed negative antiphospholipid antibodies (anti-cardiolipin and anti-β2 glycoprotein I), anti-dsDNA, and lupus anticoagulant. Complement C3 was within the normal range (Table 2).

**Table 2:** Autoimmune and TMA markers TMA: thrombotic microangiopathy; ADAMTS13: a disintegrin and metalloproteinase with thrombospondin motifs 13; C3: complement component 3

Parameter	Value	Reference Range
ADAMTS13 Activity	<1%	>67%
Anti-ADAMTS13 Antibodies	>15 U/mL Positive	Negative
Anti-cardiolipin IgG/IgM	Negative	Negative
Anti-Beta2 Glycoprotein 1 IgG/IgM	Negative	Negative
Anti-DNA	Negative	Negative
Lupus Anticoagulant	Negative	Negative
Complement C3	1.07 g/L	0.9-1.8 g/L

The patient was treated with four sessions of plasmapheresis, high-dose corticosteroids (methylprednisolone 1 g daily for three days followed by oral prednisone at 1 mg/kg/day), a single dose of rituximab (900 mg), one dose of cyclophosphamide (0.5 g/m²), and supportive measures including anti-epileptic therapy. Improvement in mental status and hematologic parameters was noted after the first session of plasmapheresis. She gradually regained full consciousness (GCS 15) and was transferred to the internal medicine department after 17 days, although segmental muscle weakness persisted - graded at 4/5 in the upper limbs and 2/5 in the lower limbs.

She remained hospitalized in the internal medicine unit for 15 additional days, during which she continued to show clinical improvement. Muscle strength gradually recovered, and biological parameters further improved. At discharge, laboratory findings showed significant recovery, with hemoglobin rising to 10.8 g/dL, platelet count to 278,000/uL, LDH down to 331 U/L, and CRP reduced to 1 mg/L. Renal function remained stable.

## Discussion

SLE is a complex autoimmune disease with a wide spectrum of clinical manifestations. Among its many complications, TTP remains rare but clinically significant due to its potential severity. TTP is a TMA characterized by severe ADAMTS13 deficiency leading to the accumulation of ultra-large von Willebrand factor (ULVWF) multimers and subsequent microvascular platelet-rich thrombi formation [1]. This results in ischemic injury, particularly affecting the CNS and kidneys.

The incidence of TTP is estimated at 3.7 per million annually, with a higher frequency in young women and individuals with autoimmune backgrounds [2]. In patients with SLE, the prevalence of TTP ranges from 1% to 4% [3,4]. The SLE-TTP presents unique diagnostic and therapeutic challenges due to overlapping features such as fever, thrombocytopenia, hemolytic anemia, renal dysfunction, and neuropsychiatric symptoms [5].

In our case, the diagnosis of immune-mediated TTP (iTTP) was confirmed by the presence of schistocytes, an ADAMTS13 activity <1%, and anti-ADAMTS13 autoantibodies. While ADAMTS13 testing is confirmatory, the PLASMIC score remains a valuable clinical tool for early recognition. This score evaluates parameters including platelet count, hemolysis markers, INR, creatinine, and the presence of active cancer or transplant history. Our patient’s score was six, categorizing her as high risk with a 72% chance of severe ADAMTS13 deficiency, thus justifying urgent plasma exchange even before confirmatory assays were available [6].

ADAMTS13 deficiency causes the accumulation of ULVWF, which promotes spontaneous platelet aggregation and the formation of microthrombi in small vessels. This leads to end-organ ischemia, particularly in the CNS, where patients often present with altered mental status, seizures, or stroke-like symptoms [1,7]. The MRI findings in our case, including leptomeningeal enhancement and periventricular white matter changes, could represent either neuropsychiatric lupus (NPSLE) or TTP-related microvascular ischemia. Distinguishing the two is critical but challenging. Petz et al. previously highlighted that CNS involvement is often prominent in both disorders and can mimic each other [8]. However, our patient's rapid neurological improvement following plasma exchange and immunosuppression supports TTP as the primary pathology.

Febrile encephalopathy with thrombocytopenia has a broad differential diagnosis. Tick-borne illnesses such as anaplasmosis, ehrlichiosis, and babesiosis can present similarly and should be considered, especially in endemic regions [9]. In particular, Anaplasma phagocytophilum has been reported to cause encephalitis and TMA-like syndromes. Blood smear review and PCR assays are essential to exclude these infections prior to initiating immunosuppression.

Patients with SLE-TTP often demonstrate a phenotype of severe thrombocytopenia, prominent CNS involvement, and relatively mild renal dysfunction, which was consistent with our case. Yue et al. found that patients with SLE-TTP and ADAMTS13 inhibitor positivity had significantly better outcomes and lower mortality than those with primary TTP [1]. This paradoxically favorable prognosis in SLE-TTP may be related to earlier diagnosis and more aggressive immunosuppressive treatment [1,4].

Our patient was promptly treated with plasma exchange and high-dose corticosteroids, resulting in rapid hematologic and neurological recovery. The cornerstone of TTP therapy remains daily therapeutic plasma exchange (TPE), which removes the inhibitor and replenishes ADAMTS13. Adjunctive immunosuppression is essential to halt autoantibody production. In refractory or relapsing TTP, rituximab has shown efficacy by depleting B-cells, with some authors advocating for its use in the first episode of iTTP when ADAMTS13 inhibitors are detected [10,11]. In our case, rituximab was initiated early due to the autoimmune nature of the TTP and concern for severe inhibitor-mediated disease.

Cyclophosphamide was also administered, given the coexisting active lupus and potential overlap with NPSLE. Although controversial, some authors suggest that cyclophosphamide may aid in treating SLE-associated TMA by targeting autoreactive lymphocytes [12]. As the patient was a young woman, fertility preservation was discussed prior to administration, and gonadoprotective measures were considered.

Eculizumab, a terminal complement inhibitor, has been reported in multiple case series to be effective in patients with SLE-associated TMA refractory to plasma exchange and steroids. Its role in cases with features of complement activation or overlap with hemolytic uremic syndrome (aHUS) is under investigation [13,14]. While our patient responded to conventional therapy, complement blockade may be considered in future relapses or refractory scenarios.

The prognosis of SLE-TTP has historically been poor, with mortality rates exceeding 50% prior to the advent of plasma exchange [3]. More recent series, however, report survival rates above 80% with prompt diagnosis and aggressive therapy [1,4,11]. Long-term follow-up is essential due to relapse risk, which can occur in up to 40% of patients [15].

This case contributes to the limited but growing literature on CNS-predominant SLE-TTP. The patient's rapid neurological recovery and hematological response underscore the importance of early recognition and timely initiation of plasma exchange and immunosuppressive therapy.

## Conclusions

The SLE-TTP is rare, yet it must be considered in lupus patients presenting with unexplained neurological symptoms, cytopenias, and laboratory evidence of hemolysis. Clinical tools such as the PLASMIC score can aid in early identification, particularly in emergency settings where ADAMTS13 testing is delayed. Nonetheless, ADAMTS13 activity measurement remains essential for definitive diagnosis and therapeutic guidance. This case demonstrates that early recognition and prompt initiation of plasma exchange, alongside corticosteroids and immunosuppressive therapy, can lead to rapid neurological improvement, resolution of hemolysis, and normalization of platelet counts. Future multicenter studies are needed to better define the clinical spectrum, treatment strategies, and long-term outcomes in patients with SLE-TTP.
